# Galvanic Vestibular Stimulation Effects on EEG Biomarkers of Motor Vigor in Parkinson's Disease

**DOI:** 10.3389/fneur.2021.759149

**Published:** 2021-11-04

**Authors:** Alireza Kazemi, Maryam S. Mirian, Soojin Lee, Martin J. McKeown

**Affiliations:** ^1^Center for Mind and Brain, Department of Psychology, University of California, Davis, Davis, CA, United States; ^2^Pacific Parkinson's Research Centre, Djavad Mowafaghian Centre for Brain Health, University of British Columbia, Vancouver, BC, Canada; ^3^Wellcome Centre for Integrative Neuroimaging (FMRIB), University of Oxford, Oxford, United Kingdom; ^4^Faculty of Medicine (Neurology), University of British Columbia, Vancouver, BC, Canada

**Keywords:** EEG, biomarker, LASSO, motor vigor, GVS, Parkinson's disease

## Abstract

**Background:** Impaired motor vigor (MV) is a critical aspect of Parkinson's disease (PD) pathophysiology. While MV is predominantly encoded in the basal ganglia, deriving (cortical) EEG measures of MV may provide valuable targets for modulation via galvanic vestibular stimulation (GVS).

**Objective:** To find EEG features predictive of MV and examine the effects of high-frequency GVS.

**Methods:** Data were collected from 20 healthy control (HC) and 18 PD adults performing 30 trials total of a squeeze bulb task with sham or multi-sine (50–100 Hz “GVS1” or 100–150 Hz “GVS2”) stimuli. For each trial, we determined the time to reach maximum force after a “Go” signal, defined MV as the inverse of this time, and used the EEG data 1-sec prior to this time for prediction. We utilized 53 standard EEG features, including relative spectral power, harmonic parameters, and amplitude and phase of bispectrum corresponding to standard EEG bands from each of 27 EEG channels. We then used LASSO regression to select a sparse set of features to predict MV. The regression weights were examined, and separate band-specific models were developed by including only band-specific features (Delta, Theta, Alpha-low, Alpha-high, Beta, Gamma). The correlation between MV prediction and measured MV was used to assess model performance.

**Results:** Models utilizing broadband EEG features were capable of accurately predicting MV (controls: 75%, PD: 81% of the variance). In controls, all EEG bands performed roughly equally in predicting MV, while in the PD group, the model using only beta band features did not predict MV well compared to other bands. Despite having minimal effects on the EEG feature values themselves, both GVS stimuli had significant effects on MV and profound effects on MV predictability via the EEG. With the GVS1 stimulus, beta-band activity in PD subjects became more closely associated with MV compared to the sham condition. With GVS2 stimulus, MV could no longer be accurately predicted from the EEG.

**Conclusions:** EEG features can be a proxy for MV. However, GVS stimuli have profound effects on the relationship between EEG and MV, possibly via direct vestibulo-basal ganglia connections not measurable by the EEG.

## Introduction

The complex ways neural activity encodes motor actions and how this can be modulated is an area of active research. While traditionally, investigation of motor control has been through hypothesis-driven approaches, with the widespread availability and sheer volume of non-invasive brain data now available, data-driven techniques can be used to complement traditional methods. The expansion of non-invasive brain stimulation (NIBS) methods has also promoted work to link brain rhythms with behavioral measures (e.g., reaction time or motor vigor [MV]), as NIBS may induce behavioral changes primarily via modulation of oscillations, as opposed to, e.g., biochemical modulations induced via pharmacotherapy. NIBS at specific phases/amplitudes/frequencies (1) or customizing stimulus parameters based on the neuroimaging data to account for individual differences (2) may ultimately lead to different behavioral effects, which could be considered as potential treatments for neurodegenerative diseases such as Parkinson's disease.

Oscillatory coupling of neural activity between the motor cortex and the basal ganglia is normally required for the execution of voluntary movements (3). The basal ganglia have diverse functionalities, including action and suppression of potentially competing actions, control of the scale of movement and related cost functions, online correction of a motor error, and motor learning (4). The dopamine deficiency seen in Parkinson's disease profoundly affects basal ganglia function, resulting in beta-band hyper synchronization (5) and an increase in the signal-dependent noise (6). Altered oscillatory/behavioral relations in Parkinson's may also lead to functional deficiencies such as impaired MV, defined as the ability to execute component movements over a range of speeds, amplitudes, and frequencies (7). The decision to move vigorously “may be thought of as an economic decision in which one spends effort to acquire a reward” (8): if a movement is deemed rewarding, one will move with increased MV, moving with shorter latency (i.e., reaction time), and faster (i.e., shorter movement time). Previous findings suggest that the quality and components of voluntary movement (e.g., velocity, accuracy, energy consumption, end-point variability, etc.) are modulated by the action execution time if the time carries a cost (8, 9). Accomplishment of a timed task can impose an implicit reward and accordingly modulate MV. In particular, bradykinesia (slowness of movement), hypokinesia (decreases in the amplitude of movements), and akinesia (poverty of movement), all key motor features of PD, are postulated to be the result of impaired movement vigor (10–12). Dopaminergic striatal activity is likely involved in value-based behavioral activation and invigoration, and a recent model of dopaminergic function suggests the dorsolateral motor striatum estimates how worthwhile it is to expend effort for the energy costs of moving (13). Increasing dopamine makes it more likely that an animal will decide it is worth spending energy to move or to move faster. As such, abnormal computation of vigor costs may be the basis of PD bradykinesia (14–17).

While MV is predominantly encoded in the basal ganglia (4), deriving (cortical) EEG measures of MV may provide valuable targets for modulation via NIBS methods such as galvanic vestibular stimulation (GVS). However, trying to map a low dimensional feature such as the presence/absence of a NIBS stimulus to another low dimensional feature such as MV is likely unsuitable for machine learning (ML) models, as this would require an impractically large number of trials to capture all sources of variability. In contrast, having an intermediate, relatively high dimensional representation of the brain state, such as the EEG, will allow first deducing the oscillatory/behavior relationships. Later on, the effects of psychosocial factors and/or NIBS on brain oscillations can then be determined. The risk of such an approach is that GVS may modulate basal ganglia structures at least in part through vestibulo-basal ganglia connections (18)—something the EEG may not be able to capture. Previous studies have demonstrated complex effects of GVS stimuli on ongoing EEG rhythms (19), with regions affected being associated with multisensory processing, likely via broadly distributed thalamocortical fibers. Thus determining the full range of cortical and subcortical areas involved in vestibular functioning and assessing the complex effects of GVS is still an active area of research and will likely include advanced models (20).

An apparent constraint in the investigation of MV markers in EEG is the risk that movement affects the recordings (21, 22). While there have been some improvements in EEG recordings during movement (e.g., in ambulatory settings), movement related artifact remains a severe issue often requiring sophisticated *post-hoc* analyses to remove artifact [e.g., (23)]. Since we are looking at specific EEG features related to vigor, we wanted to minimize the minimum amount of data manipulation to reduce artifacts. The most straightforward motor movement that would not interrupt the EEG would likely be a button press. While this would allow for evaluation of reaction time, it would not allow us to assess vigor *per se*. Squeezing a bulb through a resistance, as performed in our experiment, was the best candidate to address discussed challenges because (1) it allowed for both reaction time and movement time to be assessed, (2) it resulted in minimal head movement (and hence minimal EEG artifact), and (3) was not so effortful that PD subjects would remain fatigued after only a few trials.

**Figure 1:**
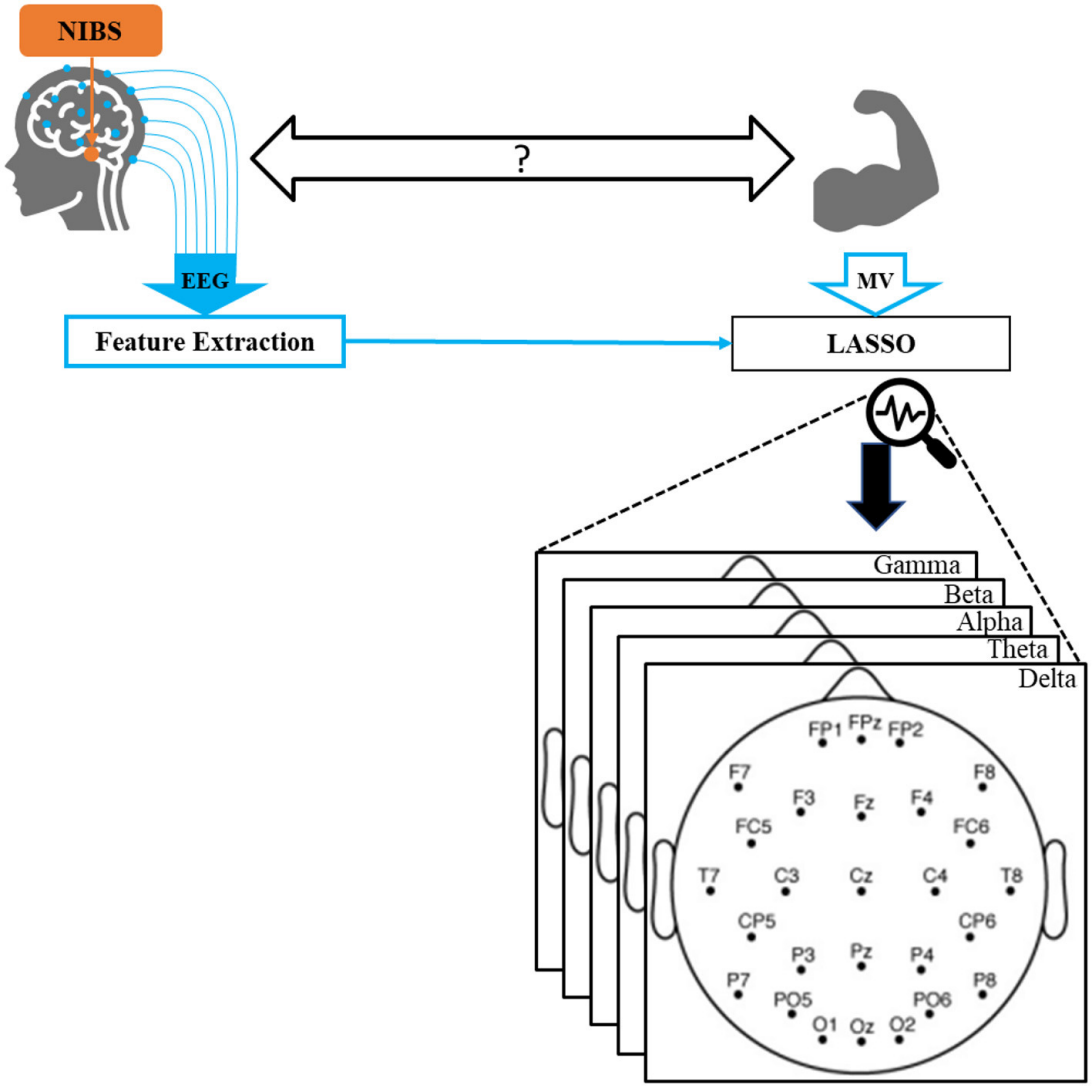
Overall schematic of our proposed method. EEG and motor vigor data were collected while participants received non-invasive brain stimulation. A comprehensive set of standard features were extracted from EEG. Utilizing LASSO, MV was predicted by a subset of features, which were finally projected back into the EEG spectral and spatial space.

In this study, we examine the relations between EEG features and MV and determine the effects of specific GVS stimuli on the EEG using LASSO regression models (24). By extracting a comprehensive set of features using LASSO models (Figure 1), we seek answers to the following questions: (a) What fraction of the MV variability can be deterministically estimated from the EEG before and during movement? (b) Which frequency sub-band(s) contribute most to accurate MV prediction? (c) Which EEG electrodes are important in terms of MV prediction? (d) What effects does GVS have on EEG/MV prediction?

## Methods

### Participants and Study Protocol

The study protocol was approved by the Clinical Research Ethics Board at the University of British Columbia (UBC), and the recruitment was conducted at the Pacific Parkinson's Research Center (PPRC). All participants gave written, informed consent before participation.

We used the same EEG and behavioral used by Lee (25). In brief, data were collected from 20 healthy controls (9 males, 67.6 ± 8.9 years) and 18 PD participants (7 males, 67.3 ± 6.5 years). Demographic and clinical characteristics of both PD and healthy controls are provided in Table 1. The experimental paradigm included a simple motor task in different blocks with 10 trials. In each block, participants received either *sham* (i.e., no) GVS stimulation or brain stimulation with different waveforms. *Sham* stimulation was performed at the beginning, and the order of the blocks with stimulation was counterbalanced between subjects.

**Table 1:** Demographic and clinical characteristics of the patients with Parkinson's disease (PD) and healthy controls (HC).

	**PD**	**HC**
Age (years), mean (sd)	68.2 (7.1)	68.6 (7.6)
Gender, n (male/female)	9/9	11/9
Disease duration (years), mean (sd)	7.4 (4.3)	-
UPDRS II, mean (sd)	14.8 (8.1)	-
UPDRS III, mean (sd)	23.3 (9.1)	-
Hoehn and yahr scale, mean (range)	1.3 (1-2)	-
Levodopa equivalent daily dose (mg), mean (sd)	635.9 (356.4)	-

*UPDRS II: Motor aspects of the experience of daily living*.

*UPDRS III: Motor symptoms*.

**Figure 2:**
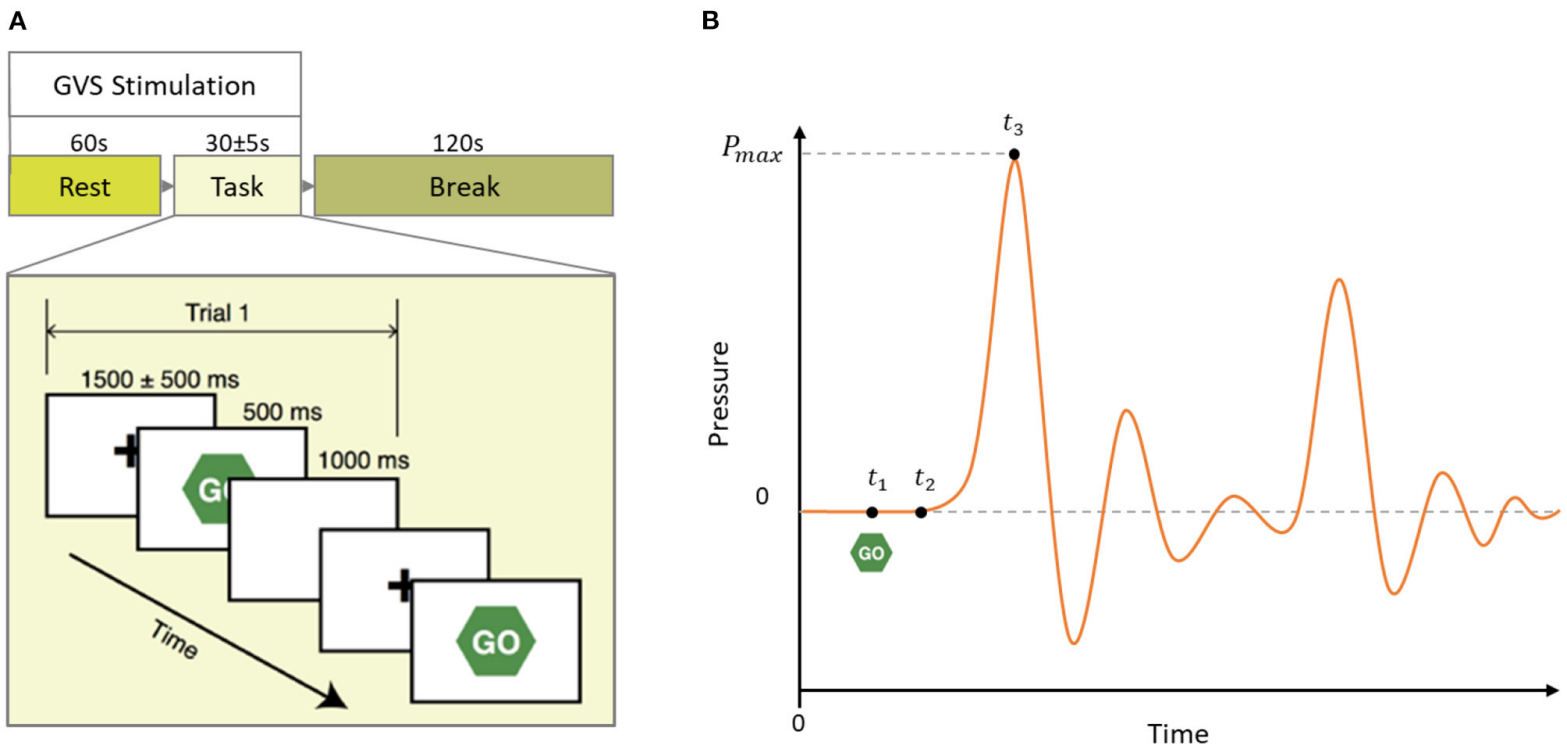
**(A)** Schematic of a block of the experimental paradigm in which 60 s rest followed by 10 task trials and 120 s break time. In each block, GVS stimulation (Sham, GVS1: 50–100 Hz, and GVS2: 100–150 Hz) was delivered during the rest and task period. **(B)** Mock pressure signal of a squeezing bulb. GO screen appeared at *t*_1_, the participant started to squeeze the bulb at *t*_2_ and reached maximum pressure at *t*_3_. Peak time is defined as *t*_3_ − *t*_1_.

During the experiment, subjects performed a simple, overlearned task. Subjects were comfortably seated in front of a computer screen and instructed to focus their gaze on a continuously displayed, fixed target for 60 sec. Then, a written instruction was given to press a key on the keyboard to start the motor task. Subjects were then instructed to respond to a visual cue (“Go”) as fast as possible by squeezing a rubber bulb. There were 10 trials each started with a 1,500 ms fixation screen jittered 500 ms followed by a 500 ms Go screen and a 1,000 ms blank screen (see Figure 2A). We formed the same size epochs of EEG signals using the last 1,000 ms of each trial time-locked at the end to the peak time of each individual subject. We chose to include EEG signals of the reaction period (up to the peak time) because we were interested in the investigation of the dynamics of neuro-modulations from both before and after task execution, which is affecting the motor vigor. We used sham (no stimulation) condition trials to characterize the dynamics of neural activities correlated with motor vigor. In addition, we pooled two other multi sine stimuli (GVS1: 50–100 Hz; GVS2: 100–150 Hz) to investigate the influence of stimulations on neural level dynamics compared to the sham condition.

### Data Collection

EEG data were recorded from 27 scalp electrodes with a sampling rate of 1 kHz using the Neuroscan SynAmp2 EEG acquisition system (Neuroscan, USA) and a standard electrode cap (64-channels Quik-Cap, Neuroscan, USA). EEG electrodes were positioned according to the international 10–20 placement standard. The reference electrode was between CPZ and CZ. The ground electrode was placed on the back of the head. The data were re-referenced to the common average. The electrodes were attached using Electro-Gel (Electrode-Cap International, USA), and impedances were kept below 15 kΩ.

### Data Analyses

#### Preprocessing

We estimated MV as the inverse of the time to reach maximum force after a “Go” signal (26) (see Figure 2B). The EEG data were first bandpass filtered between 0.5 and 45 Hz using a zero-phase finite impulse response (FIR) filter. Furthermore, we removed the artifacts using a wavelet-based filter approach (see Supplementary Material Section 1 for more information). We also performed data augmentation (27) and doubled the number of trials by downsampling by a factor of 2 to create two EEG epochs per each recorded MV. We clipped time points that were three standard deviations greater/smaller than the mean as outliers. Furthermore, after extracting 53 EEG features from each channel (see feature extraction section in the Supplementary Material Section 2), features and MVs are normalized within subjects using Z-Score scaling to bring their mean to 0 and standard deviation to 1 to minimize inter-subject variability.

#### Feature Extraction

We extracted 53 features per channel per trial, including relative spectral power, harmonic parameters, and amplitude and phase of bispectrum in frequency ranges corresponding to standard EEG channels, delta (0.5–4 hz), theta (4–8 hz), alpha-low (8–12 hz), alpha-high (12–16 hz), beta (16–32 hz), and gamma (32–45 hz). We chose this set of features to investigate and characterize the MV-related neural dynamics in the standard EEG spectral bands because standard EEG bands are well studied, and their cognitive functional correlates have been reported in the related studies. Technical details of the feature extraction section are provided in the Supplementary Material. We performed feature extraction in MATLAB, and source code is accessible online (see the code and data availability statement).

### Data Modeling

Since the data was high dimensional (53 features per 27 channels, 1,431 independent variables), we used the LASSO (least absolute shrinkage and selection operator) method (24) to find which subset of independent variables (i.e., features) gave the best linear regression model to predict MV. Since we were less interested in capturing inter-subject variabilities but rather robust features affecting MV, we performed a bootstrapping technique in 40 separate iterations on a subset of trials randomly selected from all participants in each iteration. Specifically, at each iteration, 80% of randomly selected trials were fed into LASSO algorithm to find the best regression model to predict MV, and the remaining 20% trials were used to estimate the performance of the model in predicting MV. We defined performance as the correlation between the original MV and estimated MVs by the model. We chose the correlation over the mean square error (MSE) or the mean absolute error (MAE) and/or other alternatives to best explain the dynamics and variabilities of MV and prevent a flooring effect that might affect MSE (28). We then repeated the process using only features specific to a given band (e.g., delta) to fit new LASSO models, in addition to examining the LASSO-selected coefficients using all the features to infer the spatial location, weight, and direction of the frequency-specific features that best predicted MV.

To investigate whether there were differences in MV behavioral performance between HC and PD groups in different stimulus conditions, we conducted a 2 (Disease status: HC and PD) X 3 (Stimulus: Sham, GVS1, and GVS2) mixed ANOVA, with the stimulus type varied within participants. To compare if different band-limited features affected prediction performance, we conducted a 2 (Disease status: HC and PD) by 3 (Stimulus: Sham, GVS1, and GVS2) by 7 (Bands: Broadband, Delta, Theta, Alpha-low, Alpha-high, Beta, Gamma) mixed ANOVA on average Fisher z-transformed correlations between original and estimated MVs.

All LASSO model fits and performance estimations were performed in MATLAB, and statistical analyses on performance and beta values were done in R using ANOVA and t-test comparisons. We used the Bonferroni correction to deal with multiple comparisons.

## Results

The mixed ANOVA results applied to the behavioral data showed a main effect of Stimulus type, *F*_(2,72)_ = 49.22, *p* < 0.001, η_*p*_^2^ = 0.58, such that the average MV in the *sham* condition was significantly lower (*M* = 14e-4, *SD* = 2e-4) than the average MV in both GVS1 (*M* = 15.5e-4, *SD* = 2e-4), *t*(37) = 7.74, *p* < 0.001, and GVS2 (*M* = 15.4e-4, *SD* = 2e-4), *t*(37) = 7.64, *p* < 0.001. There were no main effect or interaction effects with disease status (*p*s > 0.138).

### MV Predictability Performance

**Figure 3:**
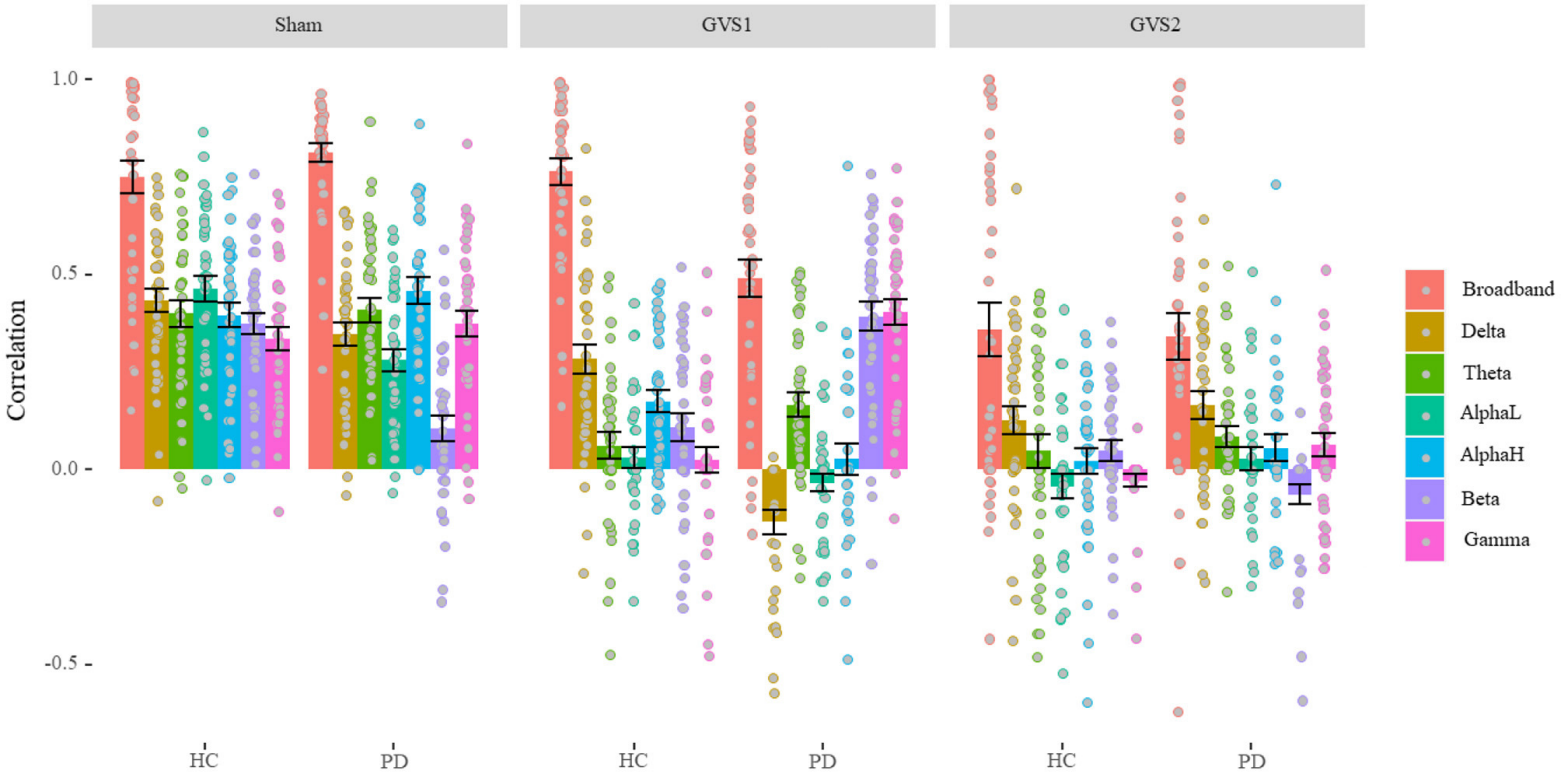
LASSO correlation performance (Model Comparison). Under GVS stimuli, the overall performance of the models is dropped except for broadband in healthy controls (HC), and beta and gamma models in the PD population under models only under GVS1. In sham conditions, band models gave almost the same level of performance except for the beta model in the PD population that gave the lowest performance. PD, Parkinson's disease; HC, healthy controls. Sham: No stimulation, GVS1: 50–100 Hz; GVS2: 100–150 Hz.

Average correlations between original and estimated MVs over 40 runs of different LASSO models are depicted separately for healthy and PD groups under each stimulus type in Figure 3 (see Supplementary Table 1 for the numerical values of the mean and standard deviation).

We found a significant main effect of Stimulus type, *F*_(2, 234)_ = 103.34, *p* < 0.001, η_*p*_^2^ = 0.47, such that average performance of predicting MV in the *sham* condition (*M* = 0.54, *SD* = 0.12) was significantly higher than that in GVS1(*M* = 0.26, *SD* = 0.11), *t*(39) = 9.56, *p* < 0.001, which in turn was significantly higher than GVS2 (*M* = 0.13, *SD* = 0.15), *t*(39) =4.33, *p* < 0.001. We also found a significant main effect of model band, *F*_(6, 1404)_ = 175.63, *p* < 0.001, η_*p*_^2^ = 0.43, such that broadband features gave the best performance (*M* = 0.98, *SD* = 0.35) overall compared to each of the other band models (*ps* < 0.042) and the alpha-low (*M* = 0.14, *SD* = 0.07) band model gave the lowest performance compared to each of the other band models (*ps* < 0.042). Average performance of delta (*M* = 0.23, *SD* = 0.09), theta (*M* = 0.21, *SD* = 0.09), alpha-high (*M* = 0.21, *SD* = 0.10), beta (*M* = 0.18, *SD* = 0.09), and gamma (*M* = 0.22, *SD* = 0.10) band models were not significantly different (*ps* > 0.061). This result is further confirmed by a significant interaction between Stimulus type and band models, *F*_(12, 1404)_ = 7.71, *p* < 0.001, η_*p*_^2^ = 0.62.

We also found a significant main effect of disease status, *F*_1,234_ = 5.64, *p* = 0.018, η_*p*_^2^ = 0.02, such that performance in PD population (*M* = 0.28, *SD* = 0.08) was significantly lower than healthy controls (*M* = 0.34, *SD* = 0.13), *t*(61.51) = 2.37, *p* = 0.021; which is confirmed by a significant interaction between disease status and band models, *F*_(6, 1404)_ = 14.98, *p* < 0.001, η_*p*_^2^ = 0.06, which is further confirmed by a significant 3-way interaction between disease, stimulus type, and band models, *F*_(12, 1404)_ = 8.29, *p* < 0.001, η_*p*_^2^ = 0.07. In each sham and GVS2 condition, broadband models were performed at the same level (*ps* > 0.191). However, broadband models significantly gave a lower performance on PD population compared to healthy in the GVS1 condition, *t*(66.84) = 5.01, *p* < 0.001. In the *sham* condition, only delta, alpha-low, and beta models had significantly different performance (lower) on PD population compared to healthy ones (*ps* < 0.03). In the GVS1 condition, delta and alpha-high models had significantly better performance in predicting MV in the healthy population (*ps* < 0.002), while theta, beta, and gamma models performed significantly better in PD population (*ps* < 0.015). Critically, under the effect of GVS1 beta and gamma models performed similarly to broadband models (*ps* > 0.31). In the GVS2 condition, beta models had significantly better performance on the healthy controls (*p* = 0.004), and gamma models performed significantly better in the PD population (*p* = 0.013). However, in the GVS2 condition, all bands had a significant performance drop, except the broadband and delta models.

To investigate the extent to which GVS1 and GVS2 affected individual feature values of the EEG in different ways and also compared to sham, we conducted a 2 (Health: HC, and PD) by 3 (Stimulus: Sham, GVS1, and GVS2) by 53 (EEG Features) mixed ANOVA. We found no main or interaction effect of stimulus (*ps* > 0.079).

### Spectral and Spatial Characterization of MV Neuro-Markers

**Figure 4:**
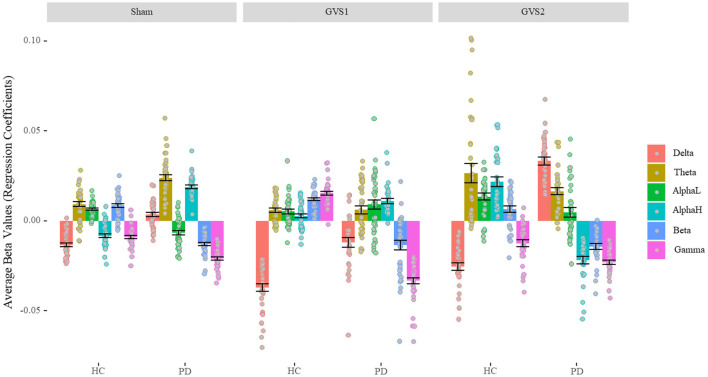
Average Beta values of BB model (Characterizing EEG bands contribution). In the healthy control group, the delta band always negatively correlated with MV, and theta, alpha-low, and beta correlated positively. In the PD population, gamma and beta always negatively correlated with MV, and theta correlated positively. PD, Parkinson's disease; HC, healthy controls. Sham: No stimulation, GVS1: 50–100 Hz; GVS2: 100–150 Hz.

Figure 4 shows the regression coefficients (beta values) of the features averaged across channels in broadband models. The numerical values are listed in Supplementary Table 2. We conducted a one-sample *t-*test to determine whether beta-values were statistically different from zero. All beta values were significantly different from zero (*ps* < 0.018) except beta values for alpha-low of PD population in the GVS2 condition (*p* = 0.100). We conducted a 2 (Disease status: HC and PD) by 3 (Stimulus: Sham, GVS1, and GVS2) by 7 (Spectral bands: Broadband, Delta, Theta, Alpha-low, Alpha-high, Beta, Gamma) mixed ANOVA and found all main and interaction effects significant (*ps* < 0.001) specifically the three-way interaction between stimulus type, health, and spectral bands, *F*_(10, 1170)_ = 52.44, *p* < 0.001, $\eta_{p}^{2}$ = 0.31, suggesting that EEG neuro-markers contribute to MV in different extents based on health status and stimulus type. Nevertheless, ignoring the absolute value across different stimuli, in the HC population, delta always negatively correlated with MV, and theta, alpha-low, and beta correlated positively, while gamma contribution under sham and GVS2 was negative and under GVS1 was positive. The PD population was more variable across different stimulus types; however, gamma and beta always negatively correlated with MV, and theta correlated positively.

**Figure 5:**
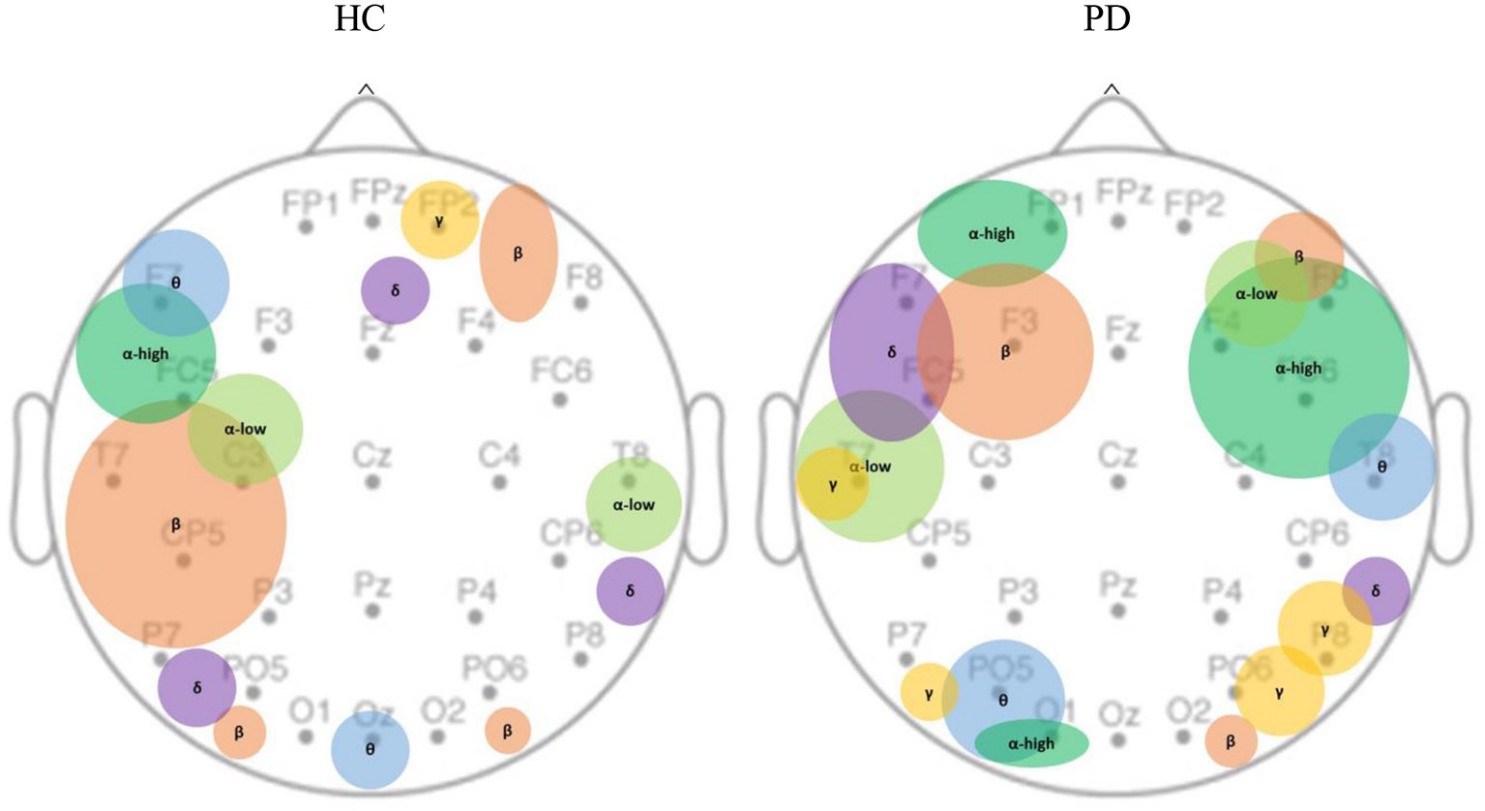
Spatial distribution of EEG bands contributed to MV under the sham condition. MV was localized over the dominant hemisphere in healthy controls as opposed to bilateral localization in PD population. In healthy controls, theta, beta, and alpha-high are primarily concentrated in the left hemisphere; gamma is only found in the mid-frontal. In PD population, delta, beta, alpha-high, alpha-low are mostly observed in the frontal areas; theta, and gamma are mostly observed in temporal, parietal, and occipital areas. PD, Parkinson's disease; HC, healthy controls. δ: Delta band; θ: Theta band; α: Alpha band; β: Beta band; γ: Gamma band.

We further investigated the spatial distribution of spectral bands in the sham condition by averaging non-zero beta values of features across different runs within each channel. Each spectral band had non-zero values only in a limited number of channels (Figure 5). We observed that the main contribution of electrodes at each EEG spectral band in the HC population was as follows: delta (PO5, P8, Fz), theta (F7, Oz), alpha-low (T8, C3), alpha-high (FC5), beta (T7, CP5, P7), gamma (FP2); and in PD group are: delta (FC5, F7), theta (PO5, T8, O1), alpha-low (T7, F4), alpha-high (FP1, FC6, F4, F8, C4, O1), beta (F3, O2, F6), gamma (P8, PO6, P7, T7).

## Discussion

Using a comprehensive set of EEG features time-locked to reaching the maximum force, we could predict MV with an accuracy of around 75% in HC and around 81% in PD. These are comparable to the prediction based on features from deep brain stimulation recordings (29), but the EEG may actually be superior to subthalamic local field potentials for movement decoding in PD (30). This suggests that, under normal conditions, cortically based EEG signals may provide sufficient information to create an MV biomarker. This is perhaps remarkable, as MV is typically assumed to be encoded in the basal ganglia, and monitoring of basal ganglia activation would normally require technologies such as fMRI. The spatial distribution of the informative channels (Figure 5, left) suggests that in controls, the beta-band features were found over the central-parietal regions, possibly related to beta band event-related desynchronization normally seen during movement (31). In controls, important channels were localized over the dominant hemisphere but were more bilateral in PD subjects, possibly related to compensatory mechanisms (32), where bilateral activity is more likely to be seen.

It is perhaps unsurprising that models that included features from all EEG sub-bands demonstrated better performance predicting MV compared to models relying on sub-band features (Figure 3). What is remarkable is that both GVS stimuli did not change the feature values themselves yet still had profound effects on both the MV in the behavioral data and on the predictability of MV from the same EEG features (Figure 3). While it is possible that the EEG features we utilized did not capture any complex effects of GVS stimuli that still influenced MV, another possibility is that the GVS stimuli are affecting non-cortical sites (not measured by the EEG) that influence MV, such as connections between the vestibular system and the basal ganglia (18). Thus, we propose that vestibular-basal ganglia connections may be central in some of GVS's effects as opposed to GVS first activating cortical regions that then influence MV.

Recent work has started to explore the role of vestibular inputs on decision-making behavior. GVS affects risk-taking behavior in healthy controls as assessed by the Balloon Analog Risk Task (BART) (33). However, this was found with left-anodal and right-cathodal GVS as opposed to the alternating currents explored here. Caloric vestibular stimulation also modulates purchase decision making (34) and vestibular stimulation has been proposed more generally to probe cognitive processes that include decision making (35). While the basis of these reward-related cognitive vestibular effects has been suggested to be an overlap between emotional circuits and vestibular regions in the cortex such as the insular and orbitofrontal cortices (34), presumably this will involve subcortical structures as well: striatal neurons encode reward independent of sensory and motor aspects (36) More work is required to delineate the cortical and subcortical contributions of GVS-related modulation of reward behavior.

The different GVS stimuli had complex effects on EEG-based MV prediction (Figure 2). Both GVS stimuli frequencies were outside the EEG band frequencies, and any changes observed outside the stimulus frequency ranges are characteristic of a non-linear system (37). The GVS stimuli used were far outside the normal physiological ranges of vestibular stimulation as would occur with, e.g., head movement, supporting the role of data-driven models that we employed here. As expected, in most cases, band-specific EEG features were less capable of predicting MV than including the features seen in all bands, as less information is available to make the prediction. However, there were some notable exceptions: with GVS1 stimuli in the PD group, the beta band predictability actually increased compared to the sham condition (Figure 3). We speculate that in PD subjects receiving GVS1 stimuli, activity along direct vestibular-basal ganglia connections allowed for the basal ganglia to again be sensitive to cortical EEG beta rhythms. The GVS2 stimuli resulted in severe degradation of MV prediction via the EEG in both controls and PD subjects. This implies an overall insensitivity of the basal ganglia to motor cortical signals, although the same stimuli still resulted in behavioral improvements in MV.

Looking at the regression coefficients in the models using all of the features (Figure 4) provides insights into the relative contributions of different EEG bands in predicting MV. In contrast to the models that only trained on band-specific features (Figure 3), the regression coefficients in Figure 4 are *relative* weights in the regression, so the weights of each EEG band can only be interpreted in the context of the other EEG bands. There are some surprises, namely that in PD subjects, both beta and gamma features were negatively correlated with MV, in the context of positive theta and high-alpha values. If beta is considered “anti-kinetic” and gamma-band activity is considered “pro-kinetic” (38), we would expect the gamma weights to be positive, not negative. This may, in part, be because the features we used included both phase and power. In controls, we found that theta, low-alpha, and beta features were associated with increasing MV, but delta, high-alpha, and gamma were associated with decreasing MV (Figure 4). In contrast, in PD subjects, theta and high-alpha were associated with increasing MV and beta and gamma (Figure 4). Although many studies have emphasized the critical role of altered beta band dynamics in PD during movement (39), gamma activity in the basal ganglia is also closely related to the coding of movement. Insufficient recruitment of fast gamma bursts during movement may underlie bradykinesia, and subthalamic gamma power correlates positively with maximal velocity (40). The above results suggest that examining specific bands for predicting MV in isolation may be misleading—individual bands must be considered in the context of other bands.

There are several limitations to our study. There are a limited number of trials which makes it hard to conduct a within-participants analysis. However, collecting a large number of trials in a motor task that require not just a simple button press, but squeezing against a resistance, can be particularly challenging in elderly and patient populations. Unlike conventional experiments that explore reward/motor behavior, in this experimental design, we had no explicit reward based on movement speed and/or accuracy. For this preliminary work, this was an explicit decision not to introduce extra confounds. We had too few trials to adequately dissociate complex aspects of decision-making processes in the EEG (i.e., monitoring reward, accuracy, and movement). Our goals were more modest here: we simply instructed people to “move as fast as they can” without additional (e.g., monetary) rewards. In addition, we only investigated standard EEG band-related features. However, defining a comprehensive set of features that can possibly capture the effects of GVS stimuli on dynamics of neural activity from EEG is not easy. Consequently, we suggest using data-driven methods like deep neural network models that can directly work with EEG signals and are not limited to hand-picked features. Nevertheless, such methods require a large amount of data to be guaranteed to find the best GVS-related features in the raw EEG signals.

In summary, despite measuring predominately cortical activity, the EEG can predict MV in both Parkinson's and control subjects. However, care must be taken to use the EEG to guide the development of GVS stimuli, as GVS affects EEG/behavioral relationships likely through vestibulo-basal ganglia pathways not measurable by the EEG.

## Data Availability Statement

The raw data supporting the conclusions of this article will be made available by the authors, without undue reservation.

## Ethics Statement

The studies involving human participants were reviewed and approved by Clinical Research Ethics Board at the University of British Columbia (UBC). The patients/participants provided their written informed consent to participate in this study.

## Author Contributions

MSM, AK, and MJM: conceptualization and writing-review and editing. MSM and AK: methodology, software, validation, formal analysis, visualization, and draft preparation. MJM: supervision and funding acquisition. SL: EEG/behavioral data collection. All authors contributed to the article and approved the submitted version.

## Funding

This work was partly supported by the John Nichol Chair in Parkinson's Research (MJM). SL was supported by Rina M. Bidin Foundation Fellowship in Research of Brain Treatment.

## Conflict of Interest

The authors declare that the research was conducted in the absence of any commercial or financial relationships that could be construed as a potential conflict of interest.

## Publisher's Note

All claims expressed in this article are solely those of the authors and do not necessarily represent those of their affiliated organizations, or those of the publisher, the editors and the reviewers. Any product that may be evaluated in this article, or claim that may be made by its manufacturer, is not guaranteed or endorsed by the publisher.
