# Supplementary material for: Galvanic Vestibular Stimulation Effects on EEG Biomarkers of Motor Vigor in Parkinson's Disease
Source: Front Neurol. 2021 Nov 4;12:759149. doi: 10.3389/fneur.2021.759149 (PMC8599939; doi:10.3389/fneur.2021.759149)
Supplement: Supplementary file 1 [file Data_Sheet_1.DOCX]

# ***Supplementary Materials***

**Table S1.** Correlation Mean values for Fig1

| Stimuli | Health | Band | M | SD |
| --- | --- | --- | --- | --- |
| Sham | HC | Broadband | 0.75 | 0.26 |
| Sham | HC | Delta | 0.43 | 0.19 |
| Sham | HC | Theta | 0.4 | 0.22 |
| Sham | HC | AlphaL | 0.46 | 0.21 |
| Sham | HC | AlphaH | 0.4 | 0.2 |
| Sham | HC | Beta | 0.37 | 0.17 |
| Sham | HC | Gamma | 0.34 | 0.19 |
| Sham | PD | Broadband | 0.81 | 0.15 |
| Sham | PD | Delta | 0.35 | 0.18 |
| Sham | PD | Theta | 0.41 | 0.2 |
| Sham | PD | AlphaL | 0.28 | 0.18 |
| Sham | PD | AlphaH | 0.45 | 0.23 |
| Sham | PD | Beta | 0.1 | 0.2 |
| Sham | PD | Gamma | 0.37 | 0.2 |
| GVS7 | HC | Broadband | 0.76 | 0.22 |
| GVS7 | HC | Delta | 0.28 | 0.23 |
| GVS7 | HC | Theta | 0.06 | 0.2 |
| GVS7 | HC | AlphaL | 0.03 | 0.16 |
| GVS7 | HC | AlphaH | 0.18 | 0.17 |
| GVS7 | HC | Beta | 0.1 | 0.22 |
| GVS7 | HC | Gamma | 0.02 | 0.19 |
| GVS7 | PD | Broadband | 0.49 | 0.3 |
| GVS7 | PD | Delta | -0.11 | 0.17 |
| GVS7 | PD | Theta | 0.17 | 0.2 |
| GVS7 | PD | AlphaL | -0.03 | 0.15 |
| GVS7 | PD | AlphaH | 0.02 | 0.21 |
| GVS7 | PD | Beta | 0.39 | 0.23 |
| GVS7 | PD | Gamma | 0.4 | 0.21 |
| GVS8 | HC | Broadband | 0.34 | 0.41 |
| GVS8 | HC | Delta | 0.13 | 0.23 |
| GVS8 | HC | Theta | 0.05 | 0.27 |
| GVS8 | HC | AlphaL | -0.04 | 0.19 |
| GVS8 | HC | AlphaH | 0.02 | 0.2 |
| GVS8 | HC | Beta | 0.04 | 0.15 |
| GVS8 | HC | Gamma | -0.02 | 0.09 |
| GVS8 | PD | Broadband | 0.34 | 0.38 |
| GVS8 | PD | Delta | 0.16 | 0.22 |
| GVS8 | PD | Theta | 0.08 | 0.16 |
| GVS8 | PD | AlphaL | 0.02 | 0.16 |
| GVS8 | PD | AlphaH | 0.05 | 0.19 |
| GVS8 | PD | Beta | -0.06 | 0.15 |
| GVS8 | PD | Gamma | 0.06 | 0.19 |

**Table S2.** Regression Coefficients Mean values for Fig2

| Stimuli | Health | Band | M | SD |
| --- | --- | --- | --- | --- |
| Sham | HC | Delta | -0.01 | 0.01 |
| Sham | HC | Theta | 0.01 | 0.01 |
| Sham | HC | AlphaL | 0.01 | 0 |
| Sham | HC | AlphaH | -0.01 | 0.01 |
| Sham | HC | Beta | 0.01 | 0.01 |
| Sham | HC | Gamma | -0.01 | 0.01 |
| Sham | PD | Delta | 0 | 0.01 |
| Sham | PD | Theta | 0.02 | 0.01 |
| Sham | PD | AlphaL | -0.01 | 0.01 |
| Sham | PD | AlphaH | 0.02 | 0.01 |
| Sham | PD | Beta | -0.01 | 0.01 |
| Sham | PD | Gamma | -0.02 | 0.01 |
| GVS7 | HC | Delta | -0.04 | 0.01 |
| GVS7 | HC | Theta | 0.01 | 0.01 |
| GVS7 | HC | AlphaL | 0.01 | 0.01 |
| GVS7 | HC | AlphaH | 0 | 0.01 |
| GVS7 | HC | Beta | 0.01 | 0 |
| GVS7 | HC | Gamma | 0.02 | 0.01 |
| GVS7 | PD | Delta | -0.01 | 0.02 |
| GVS7 | PD | Theta | 0.01 | 0.01 |
| GVS7 | PD | AlphaL | 0.01 | 0.02 |
| GVS7 | PD | AlphaH | 0.01 | 0.01 |
| GVS7 | PD | Beta | -0.01 | 0.02 |
| GVS7 | PD | Gamma | -0.03 | 0.01 |
| GVS8 | HC | Delta | -0.03 | 0.01 |
| GVS8 | HC | Theta | 0.03 | 0.03 |
| GVS8 | HC | AlphaL | 0.01 | 0.01 |
| GVS8 | HC | AlphaH | 0.02 | 0.02 |
| GVS8 | HC | Beta | 0.01 | 0.01 |
| GVS8 | HC | Gamma | -0.01 | 0.01 |
| GVS8 | PD | Delta | 0.03 | 0.01 |
| GVS8 | PD | Theta | 0.02 | 0.01 |
| GVS8 | PD | AlphaL | 0 | 0.02 |
| GVS8 | PD | AlphaH | -0.02 | 0.01 |
| GVS8 | PD | Beta | -0.01 | 0.01 |
| GVS8 | PD | Gamma | -0.02 | 0.01 |

## **Wavelet-based Artifact Rejection method**

## For artifact removal, we developed a novel method to extract an index to estimate the power of the artifact across the signal. We applied a wavelet transform with a higher power in the parts of the signal that the artifact index was closer to one and applied a wavelet with less power in other parts of the signal. To extract the artifact index, we added random Gaussian noise that has three standard deviations higher amplitude compared to EEG but less power and amplitude compared to dominant artifacts. In the timepoints that EEG had no artifact, Gaussian noise dominantly appeared in the signal instead of the original EEG signals. In the parts that EEG was affected by an artifact, the artifact remains dominant as the noise has less amplitude and power. Next, we swept the signal with a moving window and calculated the correlation between the original signal and the noise-contaminated one. To form the artifact index, we smoothed this correlation by extracting its envelope. At last, we calculated a level 4 sym6 wavelet decomposition of the original signal. We multiplied it with this artifact index which kept this wavelet decomposition signal as its original form for parts with high artifact index values and dampened the parts when the artifact index was low. Finally, we subtracted the result from the original signal to remove the artifacts and preserve the EEG signals free of artifacts.

## **EEG Feature Extraction**

For each trial and each EEG channel, 53 features are extracted, including relative spectral power, harmonic parameters, and amplitude and phase of bispectram. Feature types and groups are described in Table S1.

**Table S3**. Type and number of features extracted from each channel.

| Name | Bands | Number of Features |
| --- | --- | --- |
| Relative Spectral Power | Delta | 2 |
|  | Theta1 | 2 |
|  | Alpha-low | 2 |
|  | Alpha-high | 2 |
|  | Beta | 2 |
|  | Gamma | 1 |
| Harmonic Parameters | Delta | 3 |
|  | Theta | 3 |
|  | Alpha-low | 3 |
|  | Alpha-high | 3 |
|  | Beta | 3 |
|  | Gamma | 3 |
| Bispectram Phase | Delta | 2 |
|  | Theta | 2 |
|  | Alpha-low | 2 |
|  | Alpha-high | 2 |
|  | Beta | 2 |
|  | Gamma | 2 |
| Bispectram Amplitude | Delta | 2 |
|  | Theta | 2 |
|  | Alpha-low | 2 |
|  | Alpha-high | 2 |
|  | Beta | 2 |
|  | Gamma | 2 |

Relative powers were calculated using Welch method with hamming non-overlapping window and 4096 points discrete Fourier transform (Welch, 1967) and were computed in 10 frequency sub-bands shown in supplementary Table 2(Dumermuth & Molinari, 1987).

**Table S4**. relative power bands

| Bandwidth $\{fl,fh\}Hz$ | Sub-Band | Band |
| --- | --- | --- |
| {0.5,2.0} | Delta 1 | Delta |
| {2.0,4.0} | Delta 2 |  |
| {4.0,6.0} | Theta 1 | Theta |
| {6.0,8.0} | Theta 2 |  |
| {8.0,10.0} | AlphaHigh 1 | Alpha-Low |
| {10.0,12.0} | AlphaHigh 2 |  |
| {12.0,14.0} | AlphaLow1 | Alpha-High |
| {14.0,16.0} | AlphaLow2 |  |
| {16.0,25.0} | Beta 1 | Beta |
| {25.0,32.0} | Beta 2 |  |
| {32.0, 45.0} | Gamma 1 | Gamma |

Slow-wave index (SWI) was calculated using equation 1:

| $DSI=\frac{BSP_{Delta}}{BSP_{Theta}+BSP_{Alpha}} , TSI=\frac{BSP_{Theta}}{BSP_{Delta}+BSP_{Alpha}} , ASI=\frac{BSP_{Alpha}}{BSP_{Delta}+BSP_{Theta}}$ | (1) |
| --- | --- |

Where DSI, TSI and ASI stand for Delta Slow-wave Index, Theta Slow-wave Index and Alpha Slow-wave Index, respectively. BSP is Band Spectral Power. ASI was defined by Jobert et al. (1994) as an index for wakefulness (Jobert et al., 1994). Harmonic parameters (HP) for each band in the EEG spectrum were computed by equation 2 (Van Hese et al., 2001).

| $f_{c}=\frac{\sum_{f_{l}}^{f_{h}} fP_{xx}\left( f \right)}{\sum_{f_{l}}^{f_{h}} P_{xx}\left( f \right)}$  $f_{\sigma}=\frac{\sum_{f_{l}}^{f_{h}} \left( f-f_{c} \right)^{2}P_{xx}\left( f \right)}{\sum_{f_{l}}^{f_{h}} P_{xx}\left( f \right)}$  $S_{f_{c}}=P_{xx}\left( f_{c} \right)$ | (2) |
| --- | --- |

That, $P_{xx}$ is power spectral density function estimated for each band in EEG spectrum. Hjorth parameters provided dynamic temporal information of the EEG signals and were computed by equations 3 to 5 (Ebrahimi et al., 2008).

| $Activity=var(x)$  $Mobility=\sqrt{\frac{var\left( x^{'} \right)}{var\left( x \right)}}$  $Complexity=\sqrt{\frac{var\left( x^{''} \right).var(x)}{var\left( x^{'} \right)^{2}}}$ | (3) |
| --- | --- |
|  | (4) |
|  | (5) |

Where, $var$ is variance and $x, x^{'}$ and $x''$ are time-domain signals and their first and second-order derivatives, respectively. Skewness and kurtosis are defined in equations 6 to 8 (Zoubek et al., 2007).

| $m_{k}=\frac{1}{n}\sum_{i=1}^{n} \left( y_{i}-\bar{y} \right)^{k}$ | (6) |
| --- | --- |
| $Skewness=\frac{m_{3}}{m_{2}\times\sqrt{m_{2}}}$ | (7) |
| $Kurtosis=\frac{m_{4}}{m_{2}\times m_{2}}$ | (8) |

Bispectrum defined in equation 9 is the last feature group extracted from eachEEG epoch:

| $B\left( \omega_{1},\omega_{2} \right)=\sum_{\tau_{1}=-\infty}^{\tau_{1}=+\infty} \sum_{\tau_{2}=-\infty}^{\tau_{2}=+\infty} C\left( \tau_{1},\tau_{2} \right)e^{-j(\tau_{1}\omega_{1}+\tau_{2}\omega_{2})}$ | (9) |
| --- | --- |

Where, $C\left( \tau_{1},\tau_{2} \right)$ is the cumulant of one EEG epoch, and detailed information for computing cumulant and bispectrum can be found in (Mendel, 1991; Nikias & Raghuveer, 1987). A group of most informative data are chosen by $\phi$ and $\rho$ from bispectrum matrix $B(\omega,\phi\omega+\rho)$ and used as higher order statistical features (Swarnkar et al., 2010). In this work, 20 features are extracted from the bispectrum in two groups. 10 features are real part of bispectrum values and the others are imaginary parts corresponding to 10 frequencies $f=\{2,6,10,14,25,18,19,20,21,22\}$ Hz with$\phi=1, \rho=0$.

Finally, we decomposed the signal using SymletsWavelet at eight levels and reconstructed detail coefficients for each level. Afterwards, we computed the power of each reconstructed wavelet coefficient signal relative to the power of the original signal. These 8 levels of decomposition included more than 99% of the original signal power in almost every epoch.

# **References**

Dumermuth, G., & Molinari, L. (1987). Spectral analysis of the EEG. Some fundamentals revisited and some open problems. *Neuropsychobiology*, *17*(1–2), 85–99. https://doi.org/118345

Ebrahimi, F., Mikaeili, M., Estrada, E., & Nazeran, H. (2008). Automatic sleep stage classification based on EEG signals by using neural networks and wavelet packet coefficients. *Conference Proceedings : ... Annual International Conference of the IEEE Engineering in Medicine and Biology Society. IEEE Engineering in Medicine and Biology Society. Conference*, *2008*, 1151–1154. https://doi.org/10.1109/IEMBS.2008.4649365

Jobert, M., Schulz, H., Jähnig, P., Tismer, C., Bes, F., & Escola, H. (1994). A computerized method for detecting episodes of wakefulness during sleep based on the alpha slow-wave index (ASI). *Sleep*, *17*(1), 37–46.

Mendel, J. M. (1991). Tutorial on higher-order statistics (spectra) in signal processing and system theory: Theoretical results and some applications. *Proceedings of the IEEE*, *79*(3), 278–305.

Nikias, C., & Raghuveer, M. R. (1987). BISPECTRUM ESTIMATION: A DIGITAL SIGNAL PROCESSING FRAMEWORK. *Proceedings of the IEEE*, *75*(7), 869–891.

Swarnkar, V., Abeyratne, U., & Hukins, C. (2010). Objective measure of sleepiness and sleep latency via bispectrum analysis of EEG. *Medical and Biological Engineering and Computing*, *48*(12), 1203–1213.

Van Hese, P., Philips, W., De Koninck, J., Van de Walle, R., & Lemahieu, I. (2001). Automatic detection of sleep stages using the EEG. *2001 Conference Proceedings of the 23rd Annual International Conference of the IEEE Engineering in Medicine and Biology Society*, *2*, 1944–1947. https://doi.org/10.1109/IEMBS.2001.1020608

Welch, P. (1967). The use of fast Fourier transform for the estimation of power spectra: A method based on time averaging over short, modified periodograms. *IEEE Transactions on Audio and Electroacoustics*, *15*(2). https://doi.org/10.1109/TAU.1967.1161901

Zoubek, L., Charbonnier, S., Lesecq, S., Buguet, A., & Chapotot, F. (2007). Feature selection for sleep/wake stages classification using data driven methods. *Biomedical Signal Processing and Control*, *2*(3), 171–179. https://doi.org/10.1016/j.bspc.2007.05.005
